# Efficient Production of Multi-Layer Graphene from Graphite Flakes in Water by Lipase-Graphene Sheets Conjugation

**DOI:** 10.3390/nano9091344

**Published:** 2019-09-19

**Authors:** Noelia Losada-Garcia, Angel Berenguer-Murcia, Diego Cazorla-Amorós, Jose M. Palomo

**Affiliations:** 1 Department of Biocatalysis. Institute of Catalysis (CSIC). Marie Curie 2. Cantoblanco. Campus UAM, 28049 Madrid, Spain; n.losada@csic.es; 2 Instituto Universitario de Materiales y Departamento de Química Inorgánica, Universidad de Alicante, Apartado 99, San Vicente del Raspeig, E-03080 Alicante, Spain; a.berenguer@ua.es (A.B.-M.); cazorla@ua.es (D.C.-A.)

**Keywords:** graphene, exfoliation, lipase, graphite, biographene

## Abstract

Biographene was successfully produced in water from graphite flakes by a simple, rapid, and efficient methodology based on a bioexfoliation technology. The methodology consisted in the application of a lipase, with a unique mechanism of interaction with hydrophobic surfaces, combined with a previous mechanical sonication, to selectively generate lipase-graphene sheets conjugates in water at room temperature. The adsorption of the lipase on the graphene sheets permits to keep the sheets separated in comparison with other methods. It was possible to obtain more than 80% of graphene (in the form of multi-layer graphene) from low-cost graphite and with less damage compared to commercial graphene oxide (GO) or reduced GO. Experimental analysis demonstrated the formation of multi-layer graphene (MLG) mainly using lipase from *Thermomyces Lanuginosus* (TLL).

## 1. Introduction

Graphene is a material with excellent electrical and thermal properties, very high mechanical strength and elasticity which make it ideal for application in areas such as electronics, materials, biomedicine, biotechnology, etc. [1,2,3,4,5,6,7,8,9,10,11,12,13].

Production of graphene has been performed by scotch tape peeling [14], different chemical and thermal strategies [15,16,17,18], and solvent/surfactant-assisted exfoliation of graphite with sonication [19,20].

In particular, the synthesis of graphene by the chemical reduction of graphene oxide (GO) by the Hummers method represents at present the most scalable method for the production of few-layer graphene [15,17]. However, this technique requires very harsh and toxic conditions, which probably causes defects within the graphene sheets, compromising properties and morphology [20]. 

In this way, in recent years, strategies to synthesize graphene by a more sustainable way have been reported. The direct exfoliation of graphite to graphene in water seems to have the best reported outcome [21,22,23,24,25,26,27,28]. By this route, a few methodologies have been described to produce biographene using different small molecules (aromatic compounds, carbohydrates) [21,22,23], nanofibers [24], surfactants [25], or plant extracts [26].

In particular, the application of proteins for the exfoliation of graphite is an interesting alternative. The application of a mechanical step to create enough space between graphene layers in graphite (interlayer distance 0.33 nm) is needed for the intercalation of proteins (protein size 5–10 nm). A few examples have proven to be successful in the application of proteins [27,28,29]. However, in these cases, proteins come from animal sera or are not sufficiently pure. This is a disadvantage in order to obtain a fully selectively covered surface. Heterogeneous samples are usually obtained using these protein mixtures, where a homogeneous surface coverage should be mandatory. Another important issue is the high-efficiency exfoliation of graphite into single- or few-layered nanoplates, which remains significant and becomes the bottleneck in fundamental studies and applications of graphene. 

At present, the most specific methodology is represented by using hydrophobins, which present a hydrophobic part and another hydrophilic [30]. It is demonstrated that they can interact by the hydrophobic area on graphene. Nevertheless, these proteins are very difficult to manipulate, biochemical production is complex producing very low yields and it has been shown that mixtures of graphene and graphite are obtained following this strategy [28].

Therefore, the development of a highly selective methodology to produce a completely homogeneous modification of the graphene protein surface is mandatory. Moreover, from an economical point of view, the availability of the enzyme is critical.

**Figure 1:**
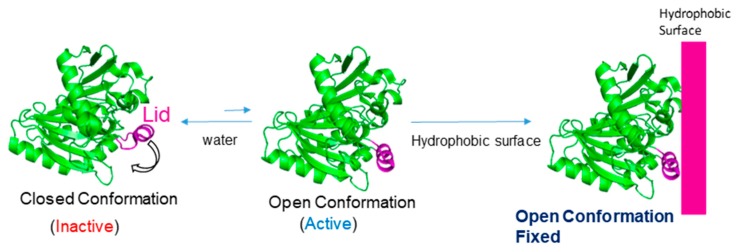
Mechanism and interaction of lipases with hydrophobic surfaces.

This way, the use of lipases can solve these drawbacks. Lipases are enzymes with a particular structure based on their natural function, fat hydrolysis. In nature, they work at the interface between oil and water [31]. They present an oligopeptide lid with an internal hydrophobic face and external hydrophilic one which is mainly in a closed conformation in homogeneous aqueous media in a certain equilibrium with a very minority open form (Figure 1). However, in the presence of hydrophobic surfaces (solid support, proteins, lipids, etc.) this equilibrium is rapidly shifted to the open conformation where the hydrophobic phase of the lid is exposed to the solvent, generating a big and exclusive hydrophobic pocket which permits to fix the enzyme in this conformation to the hydrophobic surface as an exclusive mechanism. This allows to obtain all the lipase molecules perfectly adsorbed in open conformation (Figure 1) [32,33,34,35]. 

**Figure 2:**
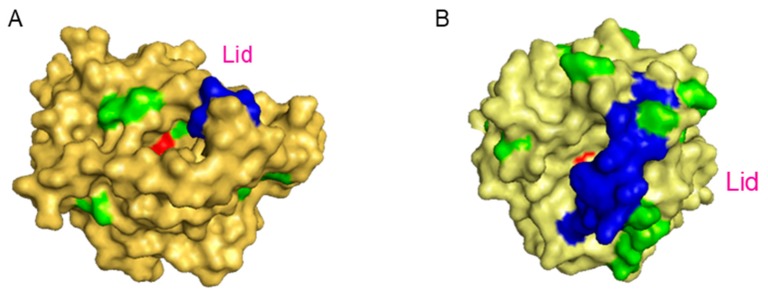
Hydrophobic area and Lid of (**A**) CAL-B, (**B**) TLL. Lid (**blue**) and Hydrophobic area (**green**).

This capacity has been demonstrated to work in the presence of different hydrophobic interfaces (detergents, solid materials, hydrophobic proteins) [32,33,34,35] (Figure 2). 

Recently a lipase interacting with graphene has been described although in all cases as a covalent immobilization technique on graphene oxide using crosslinkers [36,37].

In this work, we applied for the first time the special selective capacity of these lipases for their selective adsorption on graphene from graphite flakes. These represent a very fast, cost-efficient and sustainable methodology for the bioexfoliation of graphite to graphene. These enzymes can be homogeneously located on the graphene surface, permitting their direct production from the graphite present in a water suspension at room temperature.

This also represents a method for the specific functionalization of graphene, because we can selectively control the enzyme location, thus controlling the number of groups (amino, carboxyl) for the modification of graphene.

Furthermore, we demonstrate how the capacity of the lid has an influence on the final results and how it is possible to obtain biographene very easily on a large scale.

## 2. Materials and Methods 

### 2.1. Materials

Graphite flakes and p-nitrophenilpropionate (pNPP) were purchased from Sigma-Aldrich Co (St Louis, MO, USA). Lipase B from Candida antarctica (CAL-B) solution and lipase from Thermomyces Lanuginosus (TLL) were obtained from Novozymes (Copenhagen, Denmark). Aqueous solutions were prepared using UltraPure™ DNase/RNase-Free Distilled Water (Thermo Fisher Scientific). To recover the biohybrids, a Biocen 22 R (Orto-Alresa, Ajalvir, Spain) refrigerated centrifuge was used. 

### 2.2. Characterization Techniques

Inductively coupled plasma-optical emission spectrometry (ICP-OES) was performed in an OPTIMA 2100 DV instrument (PerkinElmer, Waltham, MA, USA). X-Ray diffraction (XRD) patterns were obtained using a Texture Analysis D8 Advance Diffractometer (Bruker, Billerica, MA, USA) with Cu Kα radiation. Transmission electron microscopy (TEM) and high-resolution TEM microscopy (HRTEM) images were obtained on a 2100F microscope (JEOL, Tokyo, Japan). Interplanar spacing in the nanostructures was calculated by using the inversed Fourier transform with the GATAN digital micrograph program (Corporate Headquarters, Pleasanton, CA, USA). Scanning electron microscopy (SEM) imaging was performed on a TM-1000 microscope (Hitachi, Tokyo, Japan). Vibra-Cell™ Ultrasonic Liquid Processors VCX130 (Sonics) was used for graphite sonication process. Raman spectra were collected at room temperature using a dispersive JASCO NRS-5100 equipment with a focal distance of 300 mm and a maximum resolution of 1 cm-1. The laser used in the experiments was a solid-state laser (532 nm) with a sample output power of 4 mW. The equipment is coupled to an MPLFLN 20 × UV objective. Prior to analysis, the Raman shift was calibrated using the 520 cm^−1^ band of a pure silicon sample. Two 60-second scans were performed for each spectrum. The obtained spectra were baseline corrected in order to remove any background fluorescence. Spectrophotometric analyses were run on a V-730 spectrophotometer (JASCO, Tokyo, Japan). Atomic force microscopy images were obtained using an NTEGRA PRIMA NT-MDT microscope. The obtained images were analyzed using the built-in software provided by the manufacturer.

### 2.3. Ultrasonication of Graphite Flakes

#### 2.3.1. Method 1: Simple Sonication of Graphite Flakes

The graphite flakes (1 g) are added together with 20 mL of distilled water to a 50 mL centrifuge tube (50 mL Falcon tube). Then, the graphite is exfoliated by alternating cycles of 5 min sonication/rest, for 30 min at an amplitude of 80%. To avoid excessive heating of the mixture, the 50 mL Falcon tube is introduced into a mixture of ice and cold acetone. This exfoliation allows the weakening of the bonds between sheets. The non-exfoliated graphite is separated from the mixture by centrifugation. The mixture was transferred to 15 mL centrifuge tubes (15 mL Falcon tubes) and centrifuged at 400 rpm for 1 min. The supernatant is carefully separated from the unfolded graphite solid. Finally, the supernatant was placed in a Petri dish and dried in the stove at 50 °C. The method allows obtaining 100 mg of Sgraphene-1, final yield of 10%.

#### 2.3.2. Method 2: Double Sonication of Graphite Flakes

The graphite flakes (1 g) are added together with 20 mL of distilled water to a 50 mL centrifuge tube (50 mL Falcon tube). Once this is done, the graphite is exfoliated by alternating cycles of 5 min sonication/rest, for 1 h at an amplitude of 80%. To avoid excessive heating of the mixture, the 50 mL Falcon tube is introduced into a mixture of ice and cold acetone. This exfoliation allows the weakening of the bonds between sheets. The non-exfoliated graphite is separated from the mixture by centrifugation. The mixture was transferred to 15 mL centrifuge tubes (15 mL Falcon tubes) and centrifuged at 400 rpm for 1 min. The black supernatant was carefully separated from the graphite solid. Finally, it was placed in a Petri dish and dried in the stove at 50 °C. The method allows obtaining 300 mg of Sgraphene-2, final yield of 30%.

### 2.4. Exfoliation of Graphite Flakes Using Lipase

#### 2.4.1. Method 3: Double Sonication of Graphite Flakes + Selective Lipase Adsorption

The graphite flakes (1 g) in 20 mL of water were sonicated as described in Method 2. After that, commercial lipase solution of TLL (0.43 mL) or commercial lipase solution of CAL-B (1 mL), in both cases offering 5 mg lipase, were directly added to that and allowed to stir for a period of time between 30 min and 1 h. The selective adsorption of the lipase was followed by measuring the supernatant using the pNPP activity assay (described below). After lipase immobilization, the suspension turned cloudy black. Then, the mixture was transferred to 15 mL Falcon centrifuge tubes and centrifuged at 400 rpm for 1 min to remove some non-exfoliated graphite. After that, the black suspension was centrifuged at 8000 rpm for 10 min and then the water was removed. One mL of acetone was added to dissolve the black powder and then this mixture was transferred to a Petri dish and dried at 50 °C for 4 h. The method allowed obtaining 800 mg of biographene-1, final yield of 80% using TLL and 600 mg, 60% yield using CAL-B.

#### 2.4.2. Method 4: Double Sonication of Graphite Flakes in the Presence of Lipase

The graphite flakes (1 g) were added to a solution of 20 mL of water to which 0.43 mL of commercial lipase was previously added in the case of TLL or 1 mL in the case of CAL-B, (enzymatic loading: 5 mgenzyme/gsupport). Then this mixture was sonicated using the aforementioned Method 2. After that, the mixture was transferred to 15 mL Falcon centrifuge tubes and centrifuged at 400 rpm for 1 min. The supernatant was carefully separated from the unfolded graphite solid. After that, the black suspension was centrifuged at 8000 rpm for 10 min and then the water was removed. One mL of acetone was added to dissolve the black powder and then this mixture was transferred to a Petri dish and dried at 50 °C for 4 h. The method allowed obtaining 500 mg of biographene-2, final yield of 50%.

### 2.5. Hydrolytic Activity Assay of pNPP

This assay was performed by measuring the increase in the absorbance at 348 nm produced by the release of p-nitrophenol in the hydrolysis of 0.4 mM pNPP in 25 mM sodium phosphate buffer at pH 7 and 25 °C. To initialize the reaction, 20 μL of lipase solution or suspension was added to 2.5 mL of substrate solution. One international unit of pNPP activity was defined as the amount of enzyme that is necessary to hydrolyze 1 μmol of pNPP per minute (IU) under the conditions described previously. 

## 3. Results

Four different methods have been performed in order to produce graphene from cheap graphite flakes. 

First, two different methods based on a mechanical force through ultrasound to the flakes of graphite were tested, one with a simple mechanical sonication (Method 1) and another with double sonication (Method 2) of graphite flakes in distilled water. After that, two materials, **Sgraphene-1** and **Sgraphene-2** were obtained. These strategies gave moderate to low yields of graphene (black suspension) of 10% to 30%, respectively. 

**Figure 3:**
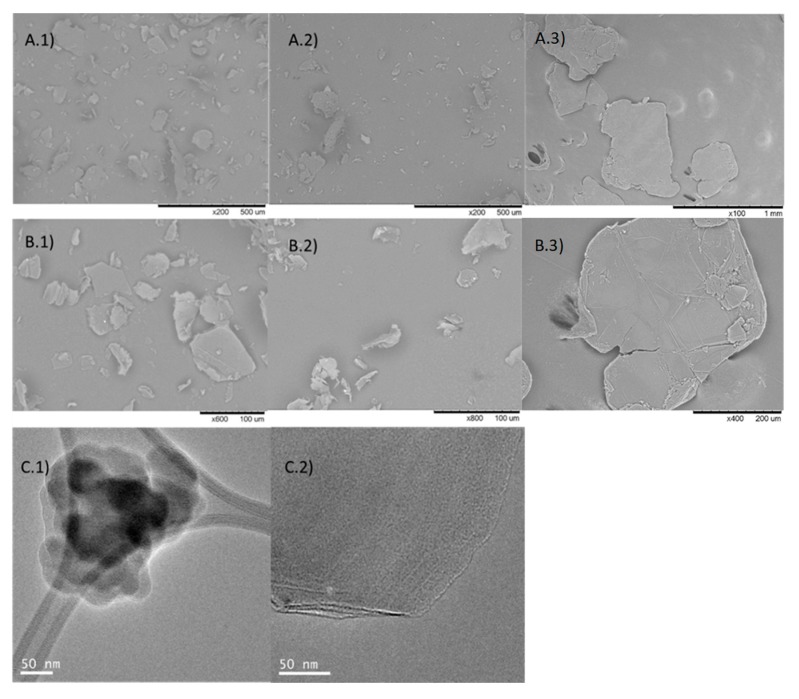
**(A,B**) SEM images. 1) **Sgraphene-1**, 2) **Sgrapehene-2**, 3) graphite flakes. (**C**) TEM images. 1) **Sgraphene-1**, 2) **Sgrapehene-2**.

If we focus on the observed differences between **Sgraphene-1** (Simple Sonication) and **Sgraphene-2** (Double sonication), for example in the SEM images, that material presented a larger flake size by using Method 1 (Figure 3A.1,B.1) than by double mechanical approach (Figure 3A.2,B.2), although in both cases clearly reduced respect to the starting commercial graphite flakes (Figure 3A,B.3). TEM analysis confirmed that **Sgraphene-1** showed a higher density solid without forming sheets (Figure 3C.1) whereas **Sgraphene-2**, a much less dense sample was observed and in which the number of layers can be appreciated (Figure 3C.2). These results seem to indicate the need for applying ultrasound for a longer time on the sample, so that progress was made on Method 2.

Considering the hydrophobic nature of graphene sheets [38] and the nature of the lipases, the next two different strategies were based on a bioexfoliation process using an enzyme combined with the sonication strategy in Method 2.

**Figure 4:**
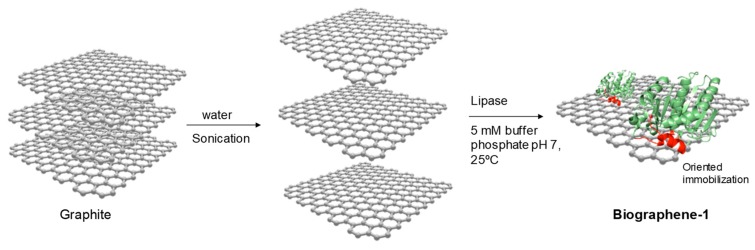
Schematic representation of **biographene-1** preparation by Method 3.

Method 3 was based on directly addition of the commercial liquid solution of lipase from *C. antarctica B* (CAL-B) or *T. lanuginosus* (TLL) on the **Sgraphene-2** (Figure 4). 

The lipase (loading of 5 mg enzyme/g support) was fully adsorbed to the solid material at room temperature. The spacing obtained previously between the graphite sheets allows the lipase to be placed between them, giving rise to hydrophobic sheet–lipase interactions (interfacial adsorption) that permit separating said sheets obtaining graphene (Figure 4). 

**Figure 5:**
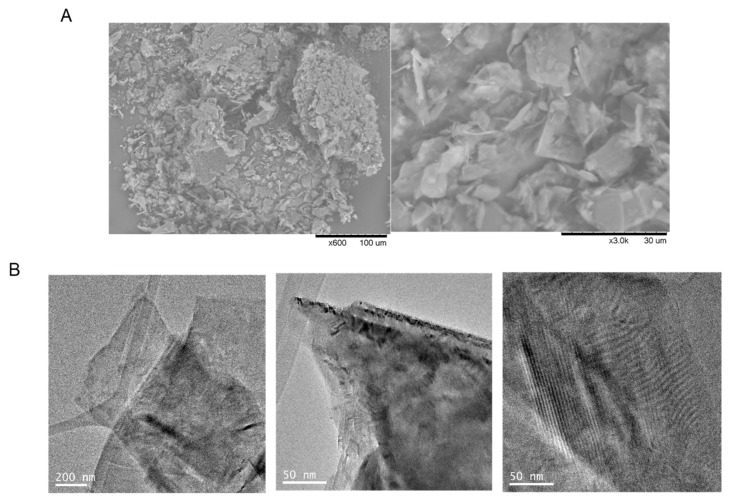
Characterization of **biographene-1**-CAL-B. (**A**) SEM Images. (**B**) TEM images.

**Figure 6:**
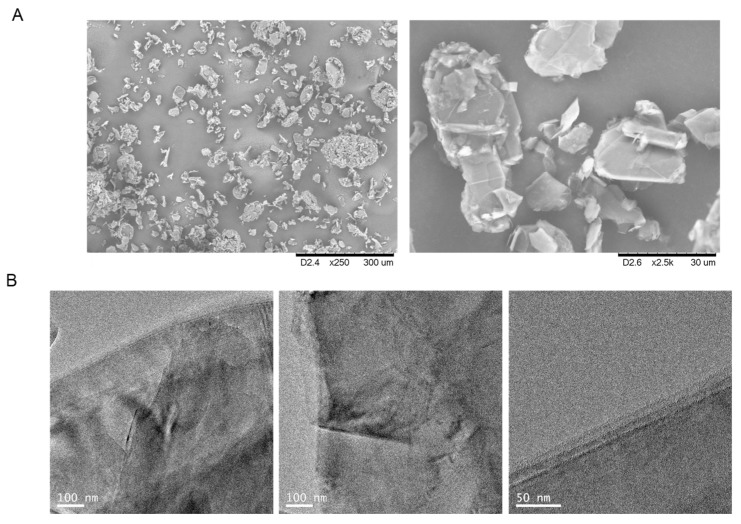
Characterization of **biographene-1-**TLL. (**A**) SEM Images. (**B**) TEM images.

The 100% immobilization was determined by measuring the enzymatic activity using the pNPP assay of the supernatant and was achieved after 30 min for TLL whereas with CAL-B 1h of incubation was necessary. Moreover, after recovery of the final **biographene-1**, the yield was higher using TLL than CAL-B, 80% against 60%. These phenomena could be explained considering the aminoacid composition and tridimensional structure of both lipases. Although both enzymes have similar molecular weight, TLL presents a much higher hydrophobic area than CAL-B (Figure 2), which has an influence on the immobilization rate of the open confirmation of the lipase on the graphene surface and also on the final interaction between enzyme and support. At first glance, the differences were evident, the aqueous suspension of graphene obtained using TLL was a homogeneous black solution while in CAL-B, although the suspension was black, a black solid slip towards the bottom was observed. These differences are also defined in SEM and TEM analysis (Figure 5 and Figure 6).

By analysis of the biographene samples with scanning electron microscopy (SEM), the method using CALB seems to generate a material with a larger flake size when compared with the method using TLL (Figure 6). The TEM images of **biographene-1** using CALB showed the presence of defects in the surface of the graphene sheets and also multiple layers superposition is seen in a disordered manner (Figure 5B). However, TEM images of **biographene-1** using TLL showed a surface with fewer defects and the number of layers present in the graphene flake was below 10, which can be observed in Figure 6B (50 nm).

**Figure 7:**
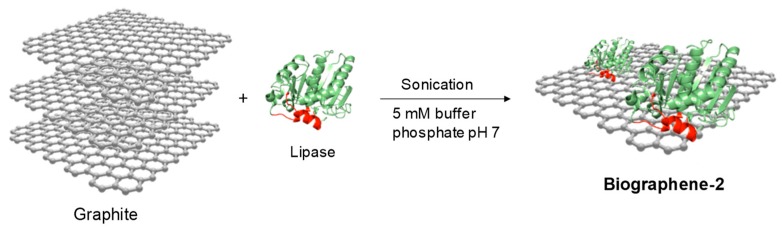
Schematic representation of **biographene-2** preparation by Method 4.

Considering these promising results, a fourth method adding the enzyme directly from the beginning (sonication plus lipase immobilization in one-pot) was attempted (Figure 7).

The lipase must be introduced between the sheets of graphene while the ultrasound weakens this interaction between sheets separating them completely. The strategy was applied using shorter and longer sonication times. In this case, also both lipases were tested.

**Figure 8:**
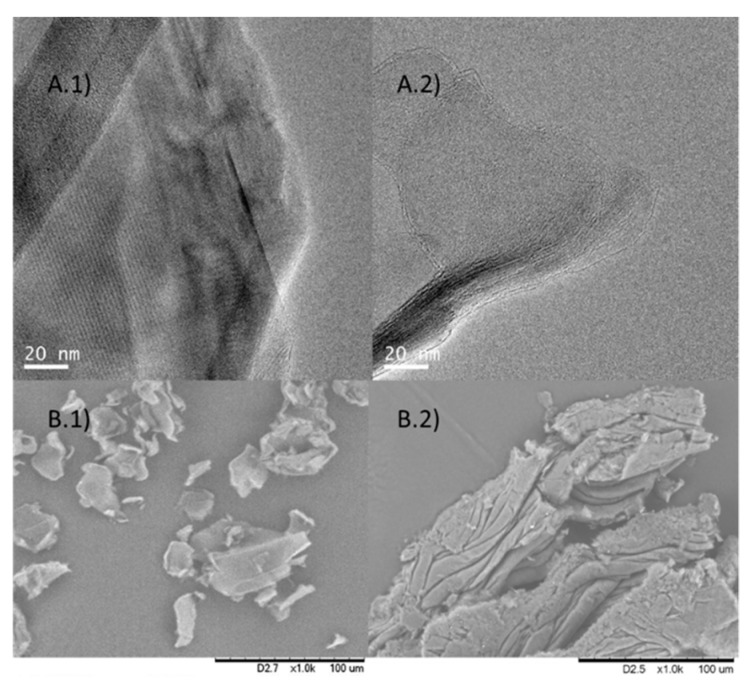
Characterization of **biographene-2**. (**A**) TEM images.1) CAL-B, 2) TLL, (**B**) SEM images. 1) CAL-B, 2) TLL.

The best results were obtained using the longest strategy for mechanical sonication described in Method 2 but in the presence of the enzyme. However, in both cases, yields of black solid around 50% were obtained. TEM analysis demonstrated differences between using one lipase or the other (Figure 8). In the case of CAL-B (Figure 8A.1) the particle size was slightly larger than in TLL (Figure 8A.2). In addition, in the SEM images (Figure 8B.1,B.2) different morphologies were observed between them. 

However, when comparing this method with the aforementioned Method 3, the sizes of the obtained aggregates were much larger than in Method 3, together with a higher density of sheets.

**Figure 9:**
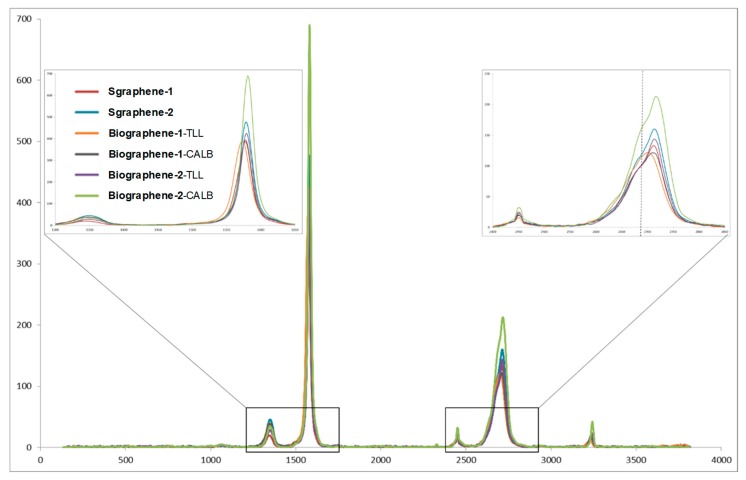
Raman spectra of the different biographene materials.

In order to determine and confirm the formation of graphene-based materials in all the cases and to know the graphene quality and the number of layers, Raman experiments were performed (Figure 9). The results established that while clear differences between methods and enzymes were found, at least one of the methods (namely Method 3 using TLL as “delaminating enzyme”) yielded interesting results (**biographene-1**-TLL). It should also be noted that the treatments applied to the different samples did not create more defects on the graphene layers, as evidenced by the comparable D band between the graphene samples and the parent graphite (see Supplementary Materials).

The **biographene-1**-TLL showed the best results according to the Raman band range of 2600–2750 cm^−1^, which clearly showed that this case a multi-layer graphene (with a number of layers between 5 and 10) is obtained, because peak at 2710 cm^−1^ is mainly present [39]. In the other cases, mixtures of graphene with a number of layers over 10 was obtained. These results reinforce the idea that better results are found using lipase with higher hydrophobicity as TLL in comparison with CAL-B. While other studies [40] have studied the splitting of the band appearing at 2450 cm^−1^ (called the G∗ or D+D′′ band) and a function of the number of graphene layers, the results have focused on samples with a number of layers <6 and thus a comparison with our results may not be applicable.

In order to corroborate the promising results hinted at by the Raman spectra, AFM images were collected (see Supplementary Materials) and analyzed for their 2D profiles in order to obtain more information on the number of layers forming the graphene flakes. The depth profiles indicated several sections in which flakes with sizes ranging from 0.2 to 1 μm had profile heights of 1.7 to 2 nm, indicative of graphene domains formed up by five and six layers, respectively, which corroborates the aforementioned Raman results.

## 4. Conclusions

We have developed for the first time a very simple, rapid, low-cost, and environmentally safe process to produce graphene sheets from very cheap graphite flakes in water and room temperature. 

The novelty consists in the application of a biomolecule, a lipase, an enzyme with a very high selectivity to interact with hydrophobic surfaces, fixing the open conformation which permits the location of lipases in open conformation uniformly distributed on the graphene sheets surface, avoiding the interactions between sheets, generating an exquisite bioexfoliation of graphite. Two different enzymes with different methods were employed and the use of *Themomyces lanuginosus* lipase (with a very high hydrophobic area) gave the best results. It was possible to produce multi-layer graphene (MLG) at 80% yield, obtaining 800 mg of graphene per gram of graphite flakes by a very simple way. Additionally, this produced better quality (i.e., less damaged) biographene than commercial GO or rGO tested.

This is very important from an industrial point of view, because economically the advantage is clear, 1 g of graphite flakes costs 3.36 Eurocents (product from SIGMA, 2.5 kg to €84), whereas 1 g of graphene oxide costs €524 (SIGMA), that means it is 15,000 times cheaper. Another advantage of this produced biographene is that it is already functionalized, because the enzyme presents a high number of carboxylic groups in the area exposed to the solvent. Therefore, this strategy could have a high potential in the application of graphene in different areas (biomedicine, biosensors, etc).

## Supplementary Materials

The following are available online at https://www.mdpi.com/2079-4991/9/9/1344/s1, Figure S1: Raman spectra of the parent graphite used in this manuscript, Figure S2: Representative AFM image (left) and inverse image (right) obtained for sample biographene-1-TLL.
