# Supplementary material for: Efficient Production of Multi-Layer Graphene from Graphite Flakes in Water by Lipase-Graphene Sheets Conjugation
Source: Nanomaterials (Basel). 2019 Sep 19;9(9):1344. doi: 10.3390/nano9091344 (PMC6781021; doi:10.3390/nano9091344)
Supplement: Supplementary file 1 [file nanomaterials-09-01344-s001.pdf]

SUPPLEMENTARY INFORMATION

Efficient production of multi-layer graphene from graphite flakes in water by lipase-graphene sheets conjugation

Noelia Losada-Garcia [1], Angel Berenguer-Murcia [2], Diego Cazorla-Amoros [2] and Jose M. Palomo [1,*]

[1] Department of Biocatalysis. Institute of Catalysis (CSIC). Marie Curie 2. Cantoblanco. Campus UAM, 28049 Madrid, Spain; n.losada@csic.es

[2] Instituto Universitario de Materiales y Departamento de Química Inorgánica, Universidad de Alicante, Apartado 99, San Vicente del Raspeig, E-03080 Alicante, Spain; a.berenguer@ua.es, cazorla@ua.es.

* Correspondence: josempalomo@icp.csic.es; Tel.: +34915854768

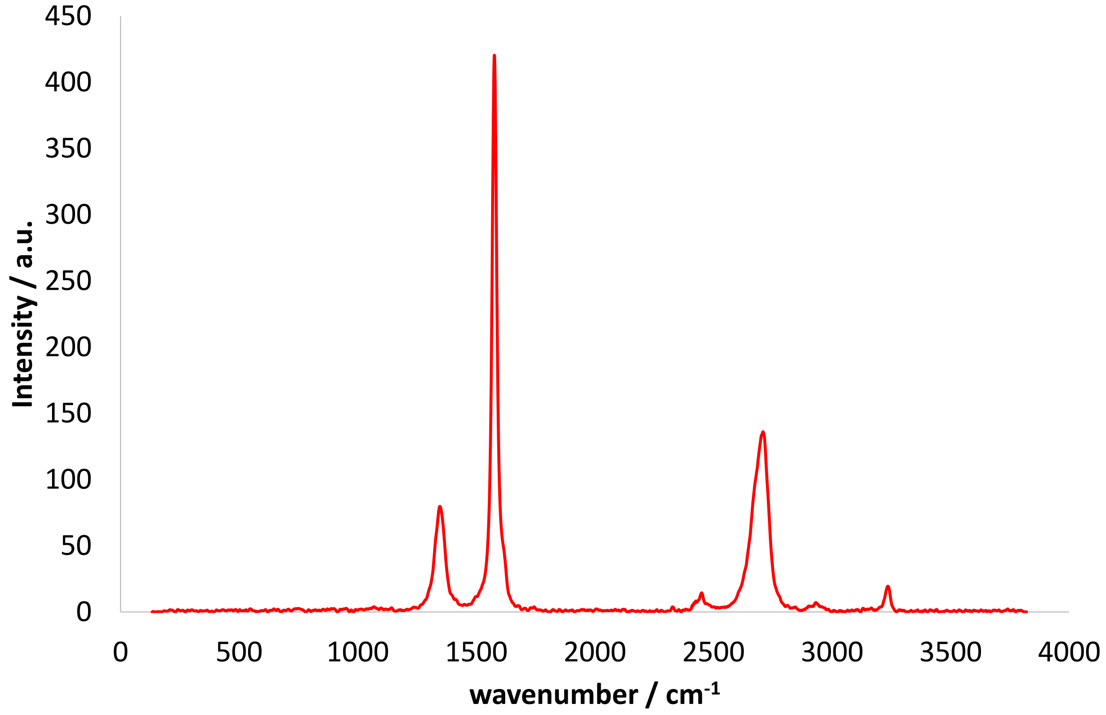

Figure 1. Raman spectra of the parent graphite used in this manuscript.

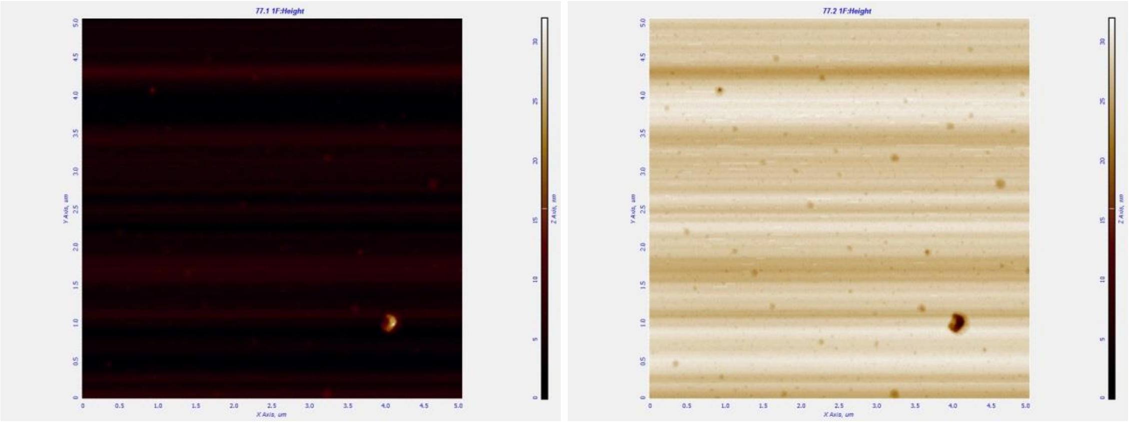

Figure 2. Representative AFM image (left) and inverse image (right) obtained for sample **biographene-1**-TLL.
